# Direct identification of *Mycobacterium abscessus* through 16S rDNA sequence analysis and a citrate utilization test: A case report

**DOI:** 10.3892/etm.2014.1705

**Published:** 2014-05-12

**Authors:** ZIYING ZOU, YUAN LIU, BING ZHU, PING ZENG

**Affiliations:** 1 Department of Microbiology and Immunology, Center of Laboratory Medicine, General Hospital of Chengdu Military Region of PLA, Chengdu 610083, P.R. China; 2 Department of Clinical Chemistry, Center of Laboratory Medicine, General Hospital of Chengdu Military Region of PLA, Chengdu 610083, P.R. China

**Keywords:** diabetes, rapidly growing mycobacteria, *Mycobacterium abscessus*, 16S rDNA sequence analysis, citrate utilization test

## Abstract

A growing number of nontuberculous mycobacteria infection cases, especially those caused by rapidly growing mycobacteria (RGM), have been reported in the past decade. Conventional methods for mycobacteria diagnosis are inefficient and easily lead to misdiagnosis. New detection methods, such as gene sequencing, have been reported but are not widely used. The aim of the present case report was to provide a quick and exact method of identifying *Myobacterium abscessus (M. abscessus)* infections. The particular case reported in this study initially manifested as hyperglycemia and papules in the right leg. Routine cultures for fungus were repeatedly negative. However, cultures of the purulent material under aerobic cultivation for five days yielded a rapidly growing, nontuberculous mycobacterium. A Ziehl-Neelsen staining of this mycobacterium revealed the presence of acid-fast bacilli that were finally identified as *M. abscessus* through 16S rDNA sequence analysis and a citrate utilization test. The current report may help other clinicians to make a quick and accurate diagnosis of RGM infection.

## Introduction

Rapidly growing mycobacteria (RGM) are characterized by rapid growth on standard media and a lack of pigmentation. They now include three clinically relevant species: *Mycobacterium fortuitum (M. fortuitum)*, *Myobacterium abscessus (M. abscessus)* and *Mycobacterium chelonae (M. chelonae)*, which are environmental microorganisms present in media including soil, bioaerosols and chlorinated water (1). RGM are able to cause chronic infections in the skin, soft tissues and lungs and these are widely reported in immunocompromised individuals (2). The present methods used for diagnosis of nontuberculous mycobacteria (NTM) are biochemical tests, quantitative polymerase chain reaction (qPCR) assays and high-performance liquid chromatography (HPLC). Due to the high homology and similarity in the clinical manifestations of *M. abscessus* with other NTM, it is difficult to identify them at the species level (3). In addition, the sequencing methods, including the ribonucleic acid polymerase beta subunit (rpoB) gene, heat-shock protein 65 gene (hsp65) gene and 16S rDNA sequencing methods, are lacking in standardized criteria for diagnosis (4). Therefore, accurate molecular techniques are urgently needed for rapid and precise diagnosis of NTM infections. In the present study, a case of a skin infection caused by *M. abscessus* is reported, which was identified by 16S rDNA sequence analysis and the citrate utilization test. Informed consent was obtained from the patient.

## Case report

A 69-year-old female was admitted to the General Hospital of Chengdu Military Region of PLA (Chengdu, China) due to swelling, nodules, ulcers and pain in the right leg. Six months previously, the patient had been impaled by a bamboo pole on the tibialis anterior of the right leg. This was followed by the gradual emergence of skin redness, suppuration and ulceration. Anti-infective medications at local clinics resulted in no clinical improvement. Three months prior to admission, the patient was noted to have a fasting blood glucose level of 18.0 mmol/l. Insulin treatment was administered and a scab formed on the wound in the leg. Approximately one month following this, several painless and erythematous subcutaneous nodules appeared on the patient’s lower right leg. Several of the nodules ulcerated and a mixture of blood and pus was exuded. There was no itching reported. The patient was diagnosed with diabetes and diabetic foot, and was given treatments for anti-infection, insulin, blood circulation activation and debridement for half a month. The blood glucose level returned to normal. When the patient was discharged, the swelling on the right leg had disappeared, although the nodules persisted and the sores had formed a crust. One month prior to admission, the number of nodules on the right leg gradually increased. There was seropurulent discharge from some of the lesions. At admission, three irregularly-shaped, dark red papules (with an approximate diameter of 1.5 cm) emerged near the right knee.

**Figure 1:**
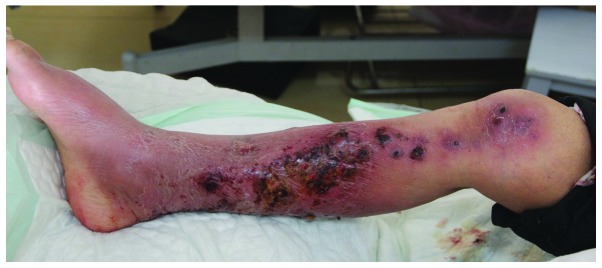
Multiple skin lesions on the right leg of the patient at admission. There was seropurulent discharge from certain lesions, and some crusts were formed.

Inspection of the lower extremities revealed multiple, painless, purple-brown colored, circular and clearly delineated nodular lesions, 2×2 cm in size, which were localized to the lower right leg and foot (Fig. 1). Crimson liquid was exuding from certain lesions and some crusts had formed. Laboratory investigations revealed that the blood glucose level was normal. No abnormalities in the biochemical and urine tests were identified. Examination of autoantibodies also revealed no abnormalities and the X-rays of the chest were unremarkable. A plain film of the right leg revealed a small area of shadow in the soft tissue area, which was considered as a foreign material. Gross pathological changes in the bones and joints were not identified. Magnetic resonance angiography (MRA) of the lower extremity vasculature revealed that stenosis was present in the peroneal artery of the lower right leg. An ultrasound scan of the lower extremity vasculature demonstrated extensive thrombosis involving the right calf muscle veins. A skin biopsy revealed signs consistent with a suppurative inflammation process in the skin, with a large number of inflammatory cells (mainly small lymphocytes) present.

**Figure 2:**
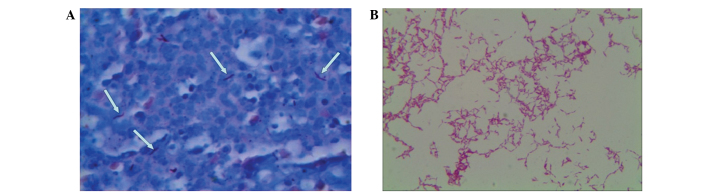
Acid-fast staining. (A) Ziehl-Neelsen staining of purulent material obtained from a draining lesion reveals multiple acid-fast bacilli (shown by arrows). (B) Direct microscopic examination of cultures of the purulent material following Ziehl-Neelsen staining was positive for acid-fast bacilli. Magnification, ×1,000.

The diagnosis of sporotrichosis and diabetes (with deep vein thrombosis) was considered. Pus was collected from the draining lesions. Fungal tests under direct microscopic examination and fungal cultures were repeatedly negative. Pus cultured on a common medium for 48 h revealed no bacterial growth. Ziehl-Neelsen staining of purulent material obtained from a draining lesion revealed the presence of multiple acid-fast bacilli (Fig. 2). Cultures of the pus on Sabouraud medium at 28°C for five days yielded a rapidly growing, nontuberculous mycobacterium. Direct microscopic examination following Ziehl-Neelsen staining was positive for acid-fast bacilli (Fig. 2). This bacterium grew well on blood, MacConkey, Sabouraud and nutrient agars at 28°C on day five. A biofilm was formed when the pus was cultured in nutrient broth for three days at 28°C. Thus, a diagnosis of ‘RGM growth’ was made.

**Figure 3:**
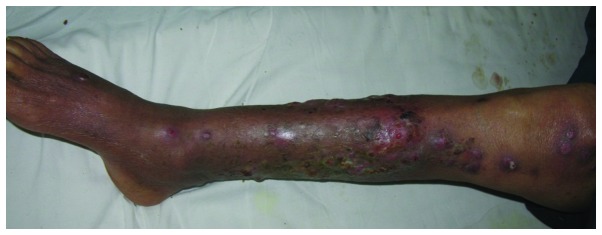
Improvement following treatment. The patient’s condition significantly improved with diminishing cutaneous lesions following a week of treatment with intravenous sulfamethoxazole 800 mg twice daily and imipenem 1 g twice daily.

The identification of this RGM isolate was further confirmed by DNA sequence analysis of 16S rDNA (corresponding to *E. coli* positions 27 to 1492 bp) using qPCR primers 27F (5′-AGAGTTTGATCCTGGCTCAG-3′) and 1492R (5′-GGYTACCTTGTTACGACTT-3′). The results revealed a sequence similarity of 100% with *M. abscessus* (GenBank accession no. NR_074427.1) and 99% with *M. chelonae* (GenBank accession no. AM884326.1). A citrate utilization test was then carried out and the result was negative. The pathogenic bacterium was ultimately identified as *M. abscessus*. Treatment was initiated with intravenous sulfamethoxazole 800 mg twice daily and imipenem 1 g twice daily in combination with debridement. One week later the condition of the patient was significantly improved with exudate cessation and diminishing cutaneous lesions (Fig. 3).

Following the identification of the microorganism as *M. abscessus* and susceptibility testing, the patient was discharged on sulfamethoxazole 800 mg twice daily by oral administration for an additional three months. Upon several follow-up visits within one year, the patient maintained a range of healthy blood sugar levels and the cutaneous lesions were gradually eliminated.

## Discussion

In the present report, the patient had a history of diabetes that was under control. To the best of our knowledge, there is no direct association between diabetes and hypoimmunity. However, certain individuals with diabetes who have a loss in glucose homeostasis may have a hypoimmunity (5). The patient was impaled by a bamboo pole and the healing of the puncture wound was delayed. Endocrinologists often do not pay much attention to the puncture wound and mistake these lesions for complications caused by diabetes itself, resulting in a misdiagnosis.

*M. abscessus* infections have similar clinical manifestations to those of other RGM infections, including *M. fortuitum* and *M. chelonae* (6). Identifying RGM to the species level is very important as their therapeutic responses are species specific. Molecular biological techniques, particularly sequencing technology, have become very powerful tools in the identification of pathogens. However, there is a difference of only 4 bp (0.37%) in the 16s rDNA between *M. abscessus* and *M. chelonae* (7). Therefore, it is insufficient to identify the pathogenic bacteria solely using 16s rDNA sequencing. Citrate utilization tests for the identification of RGM have been performed previously (*M. chelonae* is citrate positive; *M. abscessus* is citrate negative) (8). It is worth noting that 16S rDNA sequence analysis combined with a citrate utilization test is an efficient, quick and precise method of identifying the exact species of RGM. In addition, sequencing the genes of 16S–23S rDNA, IS6110, rpoB and/or hsp65 has also been suggested in order to determine the RGM species or subspecies (9,10).

In conclusion, the present case study describes a quick method for RGM identification. However, this method requires verification through the analysis of more cases and a future study to confirm the validity of the method is planned.
